# Supplementary material for: A Comparison of Mother’s Milk and the Neonatal Urine Metabolome: A Unique Fingerprinting for Different Nutritional Phenotypes
Source: Metabolites. 2022 Jan 25;12(2):113. doi: 10.3390/metabo12020113 (PMC8879468; doi:10.3390/metabo12020113)
Supplement: Supplementary file 1 [file metabolites-12-00113-s001.zip › metabolites-1560829-supplementary.pdf]

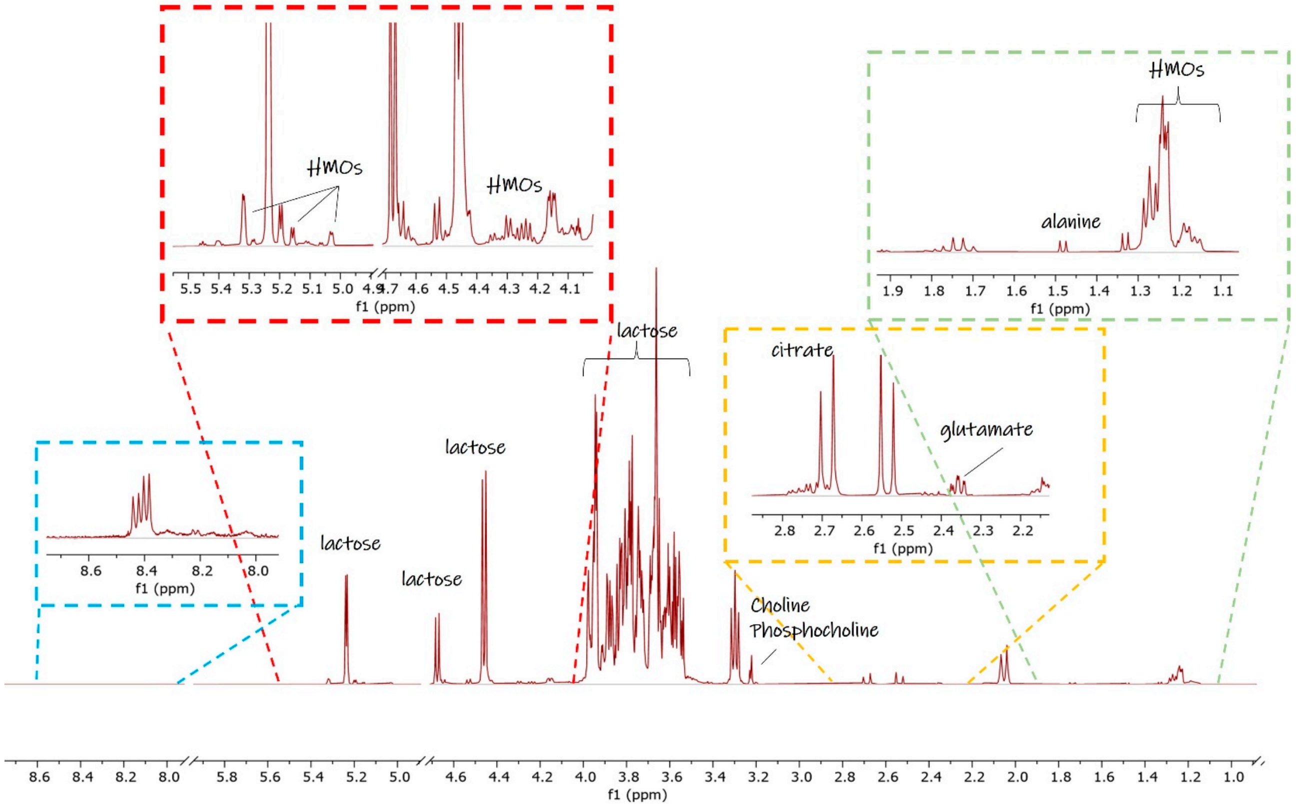


**Figure S1.** Representative $^{1}H$ NMR spectrum (500 MHz, 300 K) of human milk.

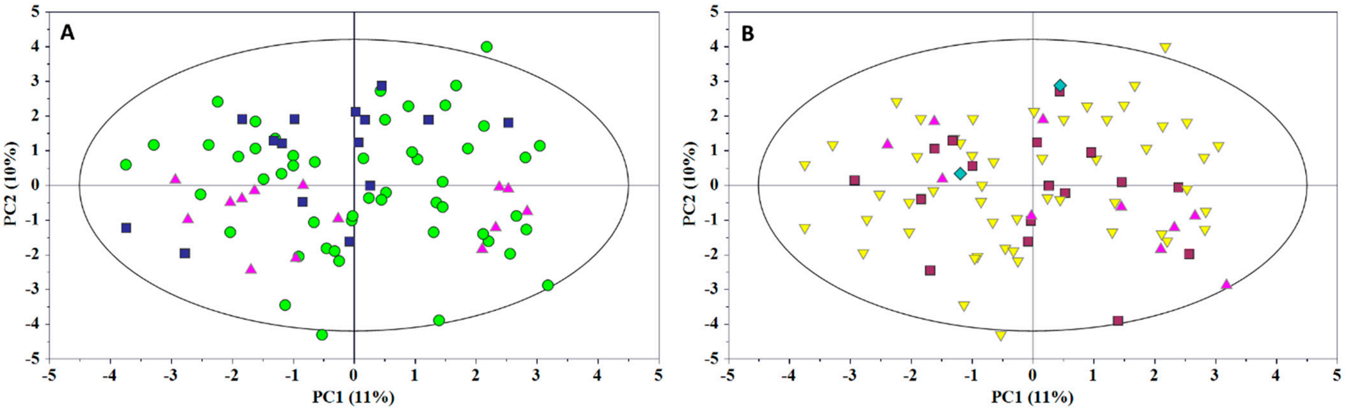


**Figure S2.** PC1 vs PC2 scores plot of urine samples. Scores are colored according to: A) the infant centile classification AGA (green circle), LGA (blue square), SGA (pink triangle) and B) the milk phenotype: $Se^{+}/Le^{+}$ (yellow inverted triangle), $Se^{+}/Le^{-}$ (pink triangle), $Se^{-}/Le^{+}$ (dark red box) and $Se^{-}/Le^{-}$ (blue diamond).
